# Terahertz Waves Trigger Apoptosis in Cutaneous Squamous Cell Carcinoma via Apoptosis-Inducing Factor Mediated Mitochondrial Pathway

**DOI:** 10.3390/cells15090810

**Published:** 2026-04-29

**Authors:** Liu Sun, Wenxia Wang, Shuocheng She, Lei Wang, Jinwu Zhao, Pandeng Hou, Mingxia He

**Affiliations:** State Key Laboratory of Precision Measuring Technology and Instruments, Tianjin University, Tianjin 300072, China

**Keywords:** 0.1 THz radiation, SCC-7 cells, mitochondrial pathway, cytochrome c, apoptosis-inducing factor

## Abstract

Background: Terahertz (THz) waves exhibit both photon-like and electron-like properties, showing emerging potential in biomedical applications. Cutaneous squamous cell carcinoma (CSCC) is one of the most common skin tumors. Studies have reported that THz waves can induce apoptosis in cancer cells or ablate tumor tissues. Our previous studies also confirmed that 0.1 THz radiation could significantly promote apoptosis in cutaneous melanoma cells, while it had no apparent effect on fibroblast viability, proliferation, migration, and apoptosis. However, the effects of 0.1 THz radiation on CSCC cells have not yet been explored. Furthermore, there remains a lack of investigation into the structural and functional effects on fibroblasts. Therefore, it is necessary to conduct a systematic study to evaluate the influence of 0.1 THz radiation on both CSCC cells and fibroblasts in order to better understand its potential therapeutic applications in the treatment of skin cancer. Purpose: This study aims to explore the biological effects of 0.1 THz radiation on SCC-7 cells and to uncover the molecular mechanisms underlying THz-induced apoptosis, as well as its potential effect on L-929 cells. Methods: Cell viability was evaluated through the CCK-8 assay, while cell cycle distribution was analyzed with the DNA content detection kit. Wound healing assays were performed to assess cell migration, and Annexin V-FITC staining was used to detect apoptosis. Caspase-3 activity was measured using the caspase-3 activity assay kit. Cell morphology was observed using the Atomic Force Microscope (AFM) and the Transmission Electron Microscopy (TEM). Alterations in membrane potential were detected with the M09 membrane potential probe kit, and intracellular Ca^2+^ levels were quantified using the Fluo-8 AM fluorescent probe. Mitochondrial permeability transition pore (mPTP) opening was assessed with the MPTP detection kit, mitochondrial membrane potential changes were measured using the JC-1 probe kit, and cellular ATP levels were measured with the enhanced ATP assay kit. Subsequently, proteomic analysis was performed. Intracellular reactive oxygen species (ROS) levels were quantified with the ROS detection kit, and cytochrome c (Cyt c) release was quantified using the mouse Cyt c ELISA kit. Apoptosis-inducing factor (AIF) expression was analyzed at both mRNA and protein levels by quantitative real-time PCR (qPCR) and Western blot. AIF expression in CSCC tissues was further evaluated based on the GSE42677 and GSE45164 databases. Finally, cyclosporin A (CsA) was used to inhibit mPTP, and in combination with the iMAC inhibitor, the Aifm1 expression and Cyt c release were examined. Results: Our results showed that THz waves significantly disrupted the membrane integrity of SCC-7 cells and induced mitochondrial structural and functional damage. This resulted in a significant increase in ROS levels and the activation of mPTP and the mitochondrial apoptosis channel (MAC). THz radiation promoted the release of Cyt c and AIF from mitochondria, triggering a noncanonical caspase-3-dependent apoptosis pathway. Notably, L-929 cells did not show significant phenotypic or apoptotic changes under the same irradiation conditions. Bioinformatics analysis of the Gene Expression Omnibus (GEO) database revealed that AIF expression was significantly altered in CSCC tissues compared to normal skin tissues. Conclusions: These findings indicated that 0.1 THz radiation effectively induced apoptosis in SCC-7 cells by triggering mitochondrial dysfunction and ROS generation, which led to the release of AIF. Furthermore, the dysregulation of AIF in CSCC tissues suggested its potential as a promising biomarker. These results provided important molecular insights into the therapeutic potential of THz radiation, particularly for the treatment of cutaneous squamous cell carcinoma.

## 1. Introduction

CSCC is one of the most common tumors among non-melanoma skin cancers originating from epidermal keratinocytes. It accounts for approximately 20% of all skin malignancies and exhibits greater aggressiveness and malignancy compared to basal cell carcinoma. The disease predominantly occurs in sun-exposed areas such as the face, scalp, neck, and dorsum of the hands. Lesions are often associated with long-standing scars, chronic ulcers, or hyperpigmentation, which are easily overlooked. Once the disease progresses to intermediate or advanced stages, the tumor will expand and metastasize, making treatment more difficult, and the patient’s risk of death also rises [1,2]. Consequently, many researchers are focusing on identifying reliable biomarkers for assessing CSCC to effectively predict patient prognosis and provide new avenues for its treatment.

THz waves exhibit both photon-like and electron-like properties, showing emerging potential in biomedical applications [3,4]. Recent studies have shown that THz radiation can induce apoptosis in cancer cells or ablate tumor tissues [5,6,7]. Our previous research also demonstrated that 0.1 THz radiation significantly promoted apoptosis in cutaneous melanoma cells [8]. The biological effects of THz waves are attributed to their strong absorption by water and their ability to resonate with the weak interactions between biomacromolecules [9,10]. Moreover, THz radiation may disrupt biological membranes and activate voltage-gated ion channels, leading to alterations in intracellular membrane structures and ion channel functions. These disruptions can compromise cellular homeostasis [11,12,13,14,15]. Such findings suggest that THz technology may offer a promising, non-invasive approach for cancer therapy and warrant further investigation in the context of CSCC treatment.

This study revealed that 0.1 THz terahertz radiation had specific biological effects on SCC-7 cells. After 2 h of irradiation, SCC-7 cells showed multiple responses: cell proliferation was significantly inhibited, the DNA synthesis (S) phase was blocked, cell migration ability decreased, and the non-classical caspase-3-dependent apoptosis pathway was activated. Further analysis suggested that THz radiation might cause cell membrane perforation, thereby reducing membrane potential and causing intracellular Ca^2+^ accumulation. This damage also led to mitochondrial structural damage, a decrease in membrane potential, the opening of permeability transition pores, and the blocking of ATP synthesis. Proteomic analysis further confirmed that THz radiation, primarily enriched in mitochondrial-related pathways, induced oxidative stress responses and thereby significantly increased ROS levels. At the molecular level, THz radiation activated non-specific mitochondrial membrane channels (the mPTP and the MAC), which promoted the release of Cyt c and AIF. Crucially, AIF may be a potential biomarker for CSCC with important implications for future diagnosis and treatment. In contrast, L-929 cells did not show significant changes under the same conditions, indicating that THz radiation may have cell-type-specific effects on SCC-7 cells.

This work first explained THz waves’ effects on cancer cells, offering a new view on physical field-biological system interactions and a translational medicine basis for developing non-invasive THz-based cancer therapies. But these findings should be further validated in clinical or more advanced animal models. Future research also should focus on optimizing THz parameters and clinically verifying AIF to promote this technology’s clinical application.

## 2. Materials and Methods

### 2.1. Cell Culture

Mouse fibroblasts (L-929, purchased from the Cell Bank of the Chinese Academy of Sciences, Serial No.: SCSP-5039, Beijing, China) were cultured in DMEM medium (Cellgro, Cat. No: 10-013-CVRC, Manassas, VA, USA) containing 10% fetal bovine serum (FBS; Newzerum, Cat. No: FBS-CE500, Christchurch, New Zealand) and 1% penicillin–streptomycin solution (Gibcao, Cat. No: 15140-122, Waltham, MA, USA). The cell line derived from a squamous cell carcinoma (SCC-7, purchased from Zhejiang Nuobio Biological Products Co., Ltd., Zhejiang, China) was cultured in RPMI 1640 medium (Cellgro, Cat. No: 10-040-CVRC, VA, USA) supplemented with 10% FBS and 1% penicillin–streptomycin solution (cell culture procedures were performed following the manufacturer’s protocols).

All cell lines used in this study have been authenticated by STR profiling and confirmed to be free of mycoplasma contamination. The cell lines were cultured under standard conditions (37 °C, 5% CO_2_, humidified atmosphere). For experiments, the seeding density was adjusted according to the plate type, with 1.5 × 10^4^ cells per well in 96-well and 1.5 × 10^5^ cells per well in 24-well plates. THz irradiation experiments were initiated when adherent cells reached approximately 80% confluence.

### 2.2. Terahertz Radiation Devices

**Figure 1:**
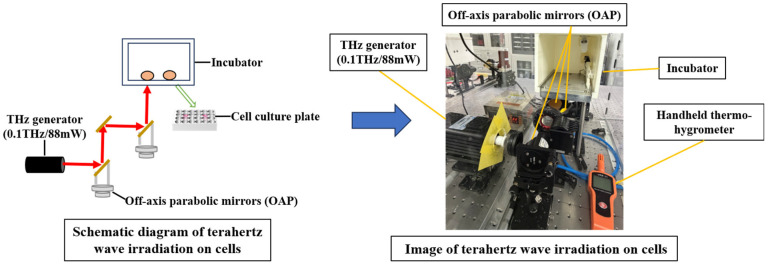
THz radiation system diagram.

In this study, a THz radiation system with a frequency of 0.1 THz and an output power of 88 mW was utilized [6]. The beam diameter was precisely adjusted to 1.5 cm to ensure full coverage of the sample wells in the exposed group. The optical setup comprised two off-axis parabolic (OAP) mirrors and a planar mirror. The emitted THz beam was initially incident on the primary OAP mirror, subsequently directed to the secondary OAP mirror, and finally reflected by the planar mirror toward a small aperture at the base of the incubator. A schematic diagram of the irradiation system is presented in Figure 1.

In the experimental setup, the control (Con) group refers to cells that were not exposed to THz radiation, but all other conditions were the same as those of the THz group. And the THz group refers to cells that were exposed to 0.1 THz radiation. Additionally, the experimental environment was maintained below 10% relative humidity using a handheld thermo-hygrometer (VICIOR, Shenzhen, China), with temperature kept constant across all trials.

### 2.3. Cell Viability Analysis

The CCK-8 assay (Solarbio, Cat. No: CA1210, Beijing, China) was employed to measure cell viability according to the manufacturer’s instructions. After THz radiation exposure, 10 μL of CCK-8 reagent (diluted in 90 μL of complete medium, total reaction system 100 μL/well) was added to each well. Blank control wells containing only CCK-8 reagent and complete medium were prepared. All samples were incubated for 1 h at 37 °C in a 5% CO_2_ humidified incubator (Yiheng, Shanghai, China) protected from light. Subsequently, absorbance measurements were taken at 450 nm employing a Multifunction Measuring Instrument (PerkinElmer, Waltham, MA, USA).

### 2.4. Cell Cycle Analysis

The DNA content measurement kit (Solarbio, Cat. No: CA1510, Beijing, China) was used to determine cell cycle distribution, with all experimental steps strictly followed according to the manufacturer’s guidelines. After THz radiation exposure, the cells were digested, centrifuged, and then suspended in phosphate-buffered saline (PBS, Cellgro, Cat. No: 21-040-CVC, VA, USA). Following repeat centrifugation, the supernatant was removed and cells were fixed overnight in 1 mL 70% ethanol at 4 °C. The following day, after washing with PBS and centrifuging again, the cells were treated with 100 μL RNase A solution at 37 °C for 30 min, followed by 400 μL propidium iodide (PI) staining solution at 4 °C for 30 min. Finally, cell cycle profiles were analyzed utilizing a flow cytometer (CytoFLEX, Beckman Coulter, Brea, CA, USA).

### 2.5. Cell Migration Analysis

The standardized scratch wound healing assay was used to assess cell migration capacity [16]. Cells were cultured to 80% confluence in a 37 °C/5% CO_2_ incubator. Monolayers were mechanically scratched using a sterile 10 μL pipette tip, followed by PBS washes to remove cellular debris. Initial wound images (t = 0 h) were captured under 4× magnification using a Nikon inverted microscope (Nikon, Tokyo, Japan). Subsequently, the cells were exposed to THz irradiation for 2 h in serum-free DMEM medium/RPMI 1640 medium. Sequential images were obtained at identical coordinates. Wound closure rates were quantified using ImageJ software (Fiji, 64-bit, Windows).

### 2.6. Cell Apoptosis Analysis

The Annexin V-FITC/PI kit was employed for apoptosis analysis (Beyotime, Cat. No: C1062S, Shanghai, China). The experimental procedures were strictly followed according to the manufacturer’s instructions. After THz radiation exposure, cells were gently washed twice with PBS and were added to 195 μL Annexin V binding buffer. Staining was conducted by adding 5 μL Annexin V-FITC and 10 μL PI working solution, followed by a 20 min dark incubation at 25 °C. Fluorescent images were captured using a fluorescence microscope (Nikon, Tokyo, Japan).

### 2.7. Caspase-3 Activity Analysis

The caspase-3 activity assay kit (Beyotime, Cat. No: C1115, Shanghai, China) was used to quantify caspase-3 enzymatic activity, in accordance with the manufacturer’s instructions. After THz radiation exposure, the cells were digested, centrifuged, and resuspended in PBS. After repeated centrifugation, cells were lysed in 50 μL lysis buffer (4 °C, 15 min) and centrifuged to collect supernatants. Protein concentrations were determined using the Bradford protein assay kit (Beyotime, Cat. No: P0006, Shanghai, China). Reaction mixtures containing 50 μL assay buffer, 10 μL cell lysate, and 10 μL Ac-DEVD-pNA substrate were incubated at 37 °C for 1 h. Absorbance at 405 nm was measured with the Multifunction Measuring Instrument. And caspase-3 activity was expressed as ΔOD/mg protein.

### 2.8. Cell Membrane Topography Analysis

AFM [17] (Park NX10, Gwacheon, Republic of Korea) was used to observe cell membrane topography. After THz radiation exposure, cells were fixed with 4% paraformaldehyde (Solarbio, Cat. No: P1110, Beijing, China) for 30 min, rinsed three times with PBS, and air-dried on slides. Pre-scan localization was performed using a Nikon inverted microscope (Tokyo, Japan). AFM imaging was conducted in tapping mode to scan. Three representative cells per sample were scanned at three cytoplasmic regions (10 μm × 10 μm). Topographical data were processed via XEI software (v4.3.4 Build22).

### 2.9. Cellular Ultrastructure Analysis

TEM [18] (JEM100 CXII, Tokyo, Japan) was used to observe cellular ultrastructure. After THz radiation exposure, cells were digested, centrifuged, and resuspended in PBS. After repeated centrifugation, the cells were fixed with 2.5% glutaraldehyde (Yuanye, Cat. No: R20515, Shanghai, China) at 4 °C for 12 h. Then, fixation was performed using a 1% osmium tetroxide/2% K_3_[Fe(CN)_6_] solution for 2 h. Next, samples were dehydrated through an ethanol gradient series. Subsequently, samples were embedded. Ultrathin sections (70 nm thickness) were obtained using a Leica UC7 ultramicrotome (Leica, Wetzlar, Germany) and doubly stained with 2% uranyl acetate (30 min) and Reynold’s lead citrate (5 min). Finally, TEM imaging was conducted.

### 2.10. Cell Membrane Potential Analysis

The M09 membrane potential probe kit (Beijing Bio-Lab Technology Co., Cat. No: HR8271, Beijing, China) was used to monitor cell membrane potential, following the manufacturer’s instructions strictly. After THz radiation exposure, the cells were washed two times with Hanks’ Balanced Salt Solution (HBSS, Gibcao, Cat. No: 14025-092, NY, USA) and then incubated in 1× M09 working solution (1 μL probe and 1 mL HBSS) for 35 min at 37 °C under dark conditions. Subsequently, the cells were stained with Hoechst 33,342 staining solution (Beyotime, Cat. No: C1027, Shanghai, China. 10 μg/mL final concentration) in the dark for 10 min. After replacing the staining solutions with HBSS, fluorescence detection was conducted using the fluorescence microscope and the multifunction measuring instrument (excitation wavelength: 488 nm, emission wavelength: 515 nm).

### 2.11. Calcium Concentration Analysis

The Fluo-8 AM fluorescent probe kit (Applygen, Cat. No: C0012, Beijing, China) was employed to measure intracellular calcium concentrations, with all experimental steps strictly followed according to the manufacturer’s guidelines. After THz radiation exposure, cells were washed twice with PBS. Each well was added with 100 μL working solution (prepared by mixing 4 μL Fluo-8 AM stock solution, 4 μL Pluronic F-127 stock solution, and 992 μL dilution buffer) and incubated under dark conditions at 37 °C for 90 min. Following incubation, the staining solution was replaced with PBS. Finally, the fluorescence microscope and the multifunction measuring instrument (excitation wavelength: 488 nm, emission wavelength: 525 nm) were used to detect intracellular calcium fluorescence.

### 2.12. MPTP Analysis

The mPTP assay kit (Abbkine, Cat. No: KTA4002, Wuhan, China) was used to assess mitochondrial membrane permeability according to the manufacturer’s instructions. After THz radiation exposure, cells were washed twice with PBS. Subsequently, 200 μL of Calcein AM staining solution (1 μL of Calcein AM and 1 mL of assay buffer) and 200 μL of fluorescence quenching solution (1 mL of Calcein AM staining solution and 10 μL of CoCl_2_) were added to separate wells. Cells were incubated at 37 °C for 30 min in the dark. After removing the staining solutions, complete medium was added, and cells were further incubated for 30 min to stabilize mitochondrial membranes. Finally, the medium was replaced with 1× assay buffer, and mitochondrial permeability was visualized using the fluorescence microscope.

### 2.13. Mitochondrial Membrane Potential Analysis

The JC-1 fluorescent probe kit (Solarbio, Cat. No: M8650, Beijing, China) was used to evaluate mitochondrial membrane potential, in accordance with the manufacturer’s instructions. After THz radiation exposure, cells were washed twice with PBS. Subsequently, 100 μL 1 × JC-1 working solution (800 μL of JC-1 dilution buffer and 200 μL of complete culture medium) was added to each well, followed by incubation at 37 °C for 30 min in the dark. After staining, the working solution was replaced with 100 μL 1 × JC-1 assay buffer. Mitochondrial membrane potential was assessed with the fluorescence microscope and the multifunction measuring instrument. For quantitative analysis, excitation/emission wavelengths were set at 490/530 nm for JC-1 monomers (green fluorescence, indicating depolarized mitochondria) and 525/590 nm for JC-1 aggregates (red fluorescence, indicating polarized mitochondria). The red/green fluorescence intensity ratio was calculated to determine changes in mitochondrial membrane potential.

### 2.14. ATP Content Analysis

The enhanced ATP assay kit (Beyotime, Cat. No: S0027, Shanghai, China). The experimental procedures were strictly followed according to the manufacturer’s instructions and were used to measure the ATP content. After THz radiation exposure, cells were rinsed twice with PBS. Subsequently, cells were lysed using lysis buffer, and the lysates were centrifuged to collect the supernatants. A portion of each sample was set aside to determine protein levels with the BCA protein assay kit. (Beyotime, Cat. No: P0012S, Shanghai, China). ATP standard and working solutions were prepared according to the manufacturer’s guidelines. Each well of the white 96-well plate was loaded with 100 μL of ATP working solution, and then 20 μL of sample or ATP standard was added. The luminometer (PerkinElmer, MA, USA) was used to measure the luminescence signals in relative light units (RLU). ATP content was determined using the standard curve and normalized per unit of total protein.

### 2.15. Proteomics Analysis

In this study, Astral 7 min DIA technology was used for mass spectrometry data acquisition, followed by protein identification and quantitative analysis [19,20]. Differentially expressed proteins were selected based on a fold change of 1.5 or 1/1.5 and a *t*-test *p*-value < 0.05. To visualize the change trends and clustering characteristics among samples, volcano plots and hierarchical clustering heatmaps were constructed. Functional annotation of the differentially expressed proteins was performed using the InterProScan software (version 6.0.0) to identify InterPro (IPR) domains. Meanwhile, an integrated analysis of metabolic pathways was performed using the Kyoto Encyclopedia of Genes and Genomes (KEGG) database. In addition, the potential protein–protein interactions between differentially expressed proteins were predicted based on the STRING database, and the interaction data were imported into Cytoscape 3.10 software to construct a protein interaction network.

### 2.16. ROS Content Analysis

The ROS assay kit (Beyotime, Cat. No: S0033S, Shanghai, China) was used to measure ROS content, following the manufacturer’s instructions strictly. Before irradiation, cells were added with 100 μL of DCFH-DA working solution (1:1000 dilution in serum-free medium) and exposed to 0.1 THz terahertz radiation for 2 h. Following irradiation, cells were incubated at 37 °C for 1 h in darkness. The staining solution was then replaced with PBS, and fluorescence was immediately detected using the Fluorescence Microscope and the Multifunction Measuring Instrument (excitation wavelength: 488 nm, emission wavelength: 525 nm).

### 2.17. Cyt c Content Analysis

The mouse Cyt c ELISA kit (Cusabio, Cat. No: CSB-E08532m, Wuhan, China) was used to quantify Cyt c levels, with all experimental procedures strictly followed according to the manufacturer’s instructions. After THz radiation exposure, cells were washed twice with PBS. Cells were lysed using lysis buffer, followed by centrifugation at 4 °C to collect the supernatant. The process involved adding 100 μL biotin–antibody working solution (diluted 1:100), incubating at 37 °C for 1 h, then replacing it with 100 μL HRP-avidin working solution (diluted 1:100), and incubating again at 37 °C for 1 h. After five washes, 90 μL TMB substrate solution was added and incubated at 37 °C in the dark for 30 min. Finally, 50 μL of stop solution was added to terminate the reaction, and the optical density at 450 nm was measured using the multifunction measuring instrument.

### 2.18. Gene Expression Analysis

After THz radiation exposure, cells were washed twice with PBS. Total RNA was extracted from cells using the TRIzol method (Abclonal, Cat. No: RK30129, Wuhan, China). The experimental procedures were strictly followed according to the manufacturer’s instructions. RNA purity was assessed using a NanoDrop One Microvolume UV-Vis Spectrophotometer (Thermo Scientific, Waltham, MA, USA) with an OD260/280 ratio of 1.8–2.0. Primers were synthesized (TaKaRa, Cat. No: hc001, Kusatsu, Japan). β-actin Forward: GTGCTATGTTGCTCTAGACTTCG, Reverse: ATGCCACAGGATTCCATACC; Heat shock protein 70 (HSP70) Forward: GTGGTGAACGACGGCGACAAG, Reverse: GCGATCTCCTTCATCTTCGTCAGC; Heat shock protein 90 (HSP90) Forward: AAACAAGGAGATTTTCCTCCGC, Reverse: CCGTCAGGCTCTCATATCGAAT; Aifm1 Forward: CTGGATGTAAGAGGCAACATGG, Reverse: CCGCCGATAACTGTAATTGACT), and reverse transcription was performed using the GoScript™ Reverse Transcriptase Kit (Promega, Cat. No: A5003, Madison, WI, USA). QPCR was performed using the GoTaq qPCR Master Mix Kit (Promega, Cat. No: A6001, WI, USA) on a LightCycler^®^ 96 System (Roche, Basel, Switzerland). Thermal cycling conditions were as follows: initial denaturation at 95 °C for 10 min, followed by 40 cycles of 95 °C for 15 s and 60 °C for 1 min. Relative gene expression levels were calculated using the 2^−ΔΔCt^ method, normalized to β-actin as an internal control.

### 2.19. Proteins Expression Analysis

The expression of AIF (MCE, Cat. No: HY-P80006, Monmouth Junction, NJ, USA. The experimental procedures were strictly followed according to the manufacturer’s instructions and were assessed using the Western blot. To begin with, total protein was extracted, and its concentration was determined with a BCA protein assay kit. Subsequently, a 10% separation gel and a stacking gel were prepared. A total of 15 μg of protein sample was loaded, and the electrophoresis voltage was set at 80 V and 120 V for electrophoresis. Following electrophoretic separation, proteins were transferred onto a PVDF membrane at a constant current of 300 mA for 1 h. The membrane was then incubated in a blocking buffer containing 5% non-fat milk for 1 h. Next, the membrane was probed with primary antibodies diluted in blocking buffer (GAPDH 1:10,000, MCE, Cat. No: HY-P80137; AIF 1:1000) and kept at 4 °C for overnight incubation. After washing, the corresponding secondary antibodies (1:10,000, Jackson, PA, USA) were applied for 1 h at room temperature. The protein bands were finally visualized using a chemiluminescent imaging system (Monad, Suzhou, China).

### 2.20. GEO Data

Two transcriptome datasets associated with CSCC, namely GSE42677 [21] and GSE45164 [22] (Tables S1 and S2), were retrieved from the GEO database. AIF expression profiles were subsequently extracted from these datasets and subjected to uniform normalization and analysis.

The GEO data were accessed in compliance with the data use policies. Given that the data used were pre-existing and fully de-identified, the study was deemed exempt from institutional review board (IRB) approval.

### 2.21. Statistical Analysis

Statistical analysis was conducted using either parametric or non-parametric tests based on the distribution characteristics of the data, and statistical inferences were made at the 95% confidence level. First, normality and homogeneity of variances were assessed. If the data met the assumptions of normality and variance homogeneity, independent samples *t*-tests were used for comparisons between two groups. Post hoc tests were subsequently performed when ANOVA results were significant. For data that did not follow a normal distribution or showed heterogeneous variances, the Mann–Whitney U test (two groups) was employed for analysis. All experimental measurements were based on three or more independent replicates (N ≥ 3) and were presented as the Mean ± SD. Statistical analyses were performed using GraphPad Prism 8.0.2, and differences were considered significant at *p* < 0.05 (* *p* < 0.05, ** *p* < 0.01, *** *p* < 0.001).

## 3. Results

### 3.1. Terahertz Radiation-Induced Cell Death of SCC-7 Cells

**Figure 2:**
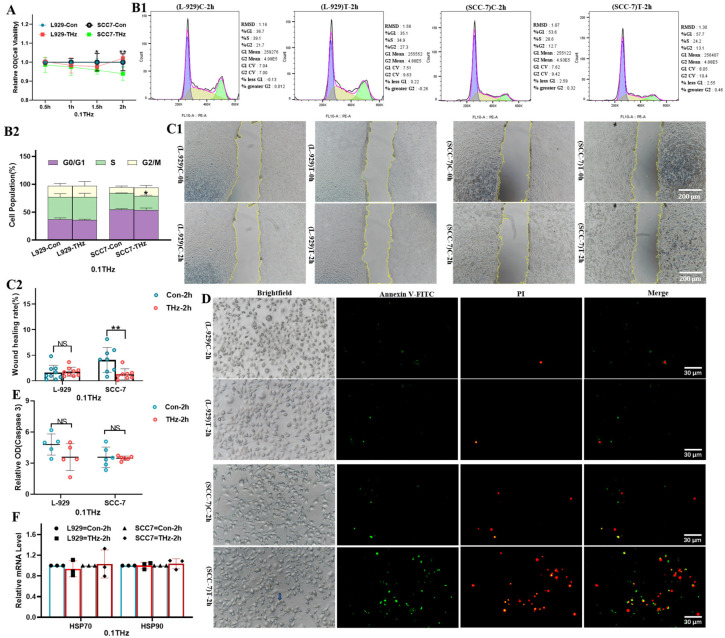
THz radiation-induced cell death in SCC-7 cells. (**A**): Viability analysis of L-929 and SCC-7 cells (N = 7). Compared with the SCC-7 cells control group, the 1.5 h and 2 h irradiated groups showed statistically significant differences (* *p* = 0.035 for 1.5 h, ** *p* = 0.009 for 2 h). (**B**): Cell cycle analysis of L-929 and SCC-7 cells. (**B1**): Flow cytometry results; (**B2**): Data analysis. (N = 3, * *p* = 0.012 for S phase). (**C**): Migration analysis of L-929 and SCC-7 cells. (**C1**): Scratch experiment image. (**C2**): Data analysis. (N = 8, ** *p* = 0.010, scale bar = 200 μm). (**D**): Apoptosis analysis of L-929 and SCC-7 cells (N = 3, scale bar = 30 μm; the blue arrows indicate apoptotic cells). (**E**): Caspase-3 activity analysis of L-929 and SCC-7 cells (N = 6, the result was not statistically significant, NS). (**F**): The expression of HSP70 and HSP90 (N = 3, Not Significant, NS).

0.1 THz terahertz radiation exhibited an inhibitory effect on SCC-7 cells. As shown by the cell viability assay, SCC-7 cells exposed for 1.5 h and 2 h demonstrated a significant reduction in viability (*p* < 0.05 or *p* < 0.01), whereas L-929 cells showed no significant changes under identical treatment conditions (Figure 2A, *p* > 0.05). Cell cycle analysis further revealed a reduction in the S phase of SCC-7 cells (Figure 2B, *p* < 0.05), indicating a possible inhibition of DNA synthesis or cell proliferation. In the migration capability assessment, THz radiation markedly suppressed the migration rate of SCC-7 cells (Figure 2C, *p* = 0.01). Although FITC/PI double staining showed enhanced apoptotic fluorescence (Figure 2D), no significant increase in caspase-3 activity was observed (Figure 2E, *p* > 0.05), suggesting potential involvement of a non-canonical apoptotic pathway. Simultaneously, the expression levels of HSP70 and HSP90 remained stable before and after irradiation, suggesting THz radiation did not induce significant thermal changes (Figure 2F, *p* > 0.05). It is worth noting that no significant changes were observed in L-929 cells in the aforementioned tests, highlighting the differential response between cell types.

### 3.2. Terahertz Radiation-Altered the Membrane Structure of SCC-7 Cells

**Figure 3:**
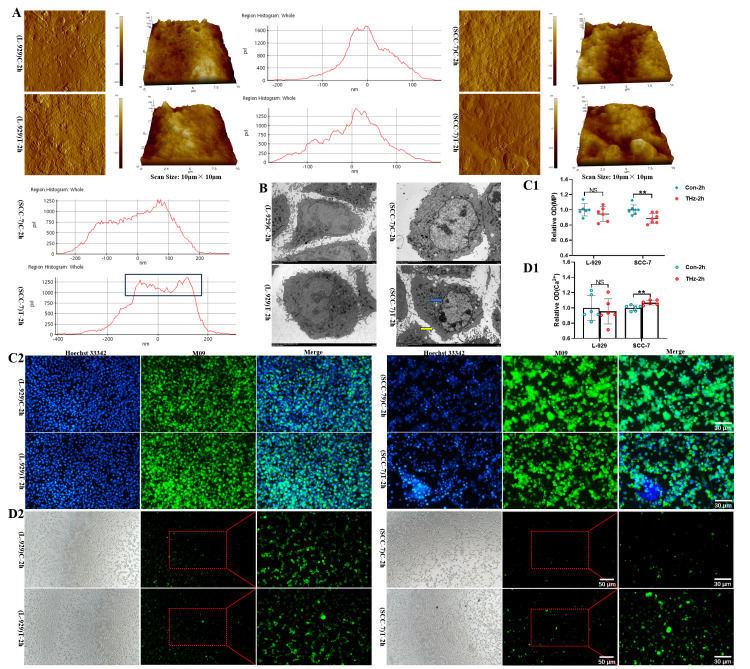
THz radiation altered the membrane structure of SCC-7 cells. (**A**): Cell membrane topography analysis of L-929 and SCC-7 cells (scan size = 10 μm × 10 μm; the black squares represented the histogram of the local membrane protrusion region). (**B**): Cellular ultrastructure analysis of L-929 and SCC-7 cells (yellow arrow indicates the cell membrane and blue arrow indicates the nucleus; scale bar = 5 μm). (**C**): Analysis of cell membrane potential in L-929 and SCC-7 cells. (**C1**): Fluorescence images. (**C2**): Data analysis. (N = 7, ** *p* = 0.007; scale bar = 30 μm). (**D**): Analysis of Ca^2+^ levels in L-929 and SCC-7 cells. (**D1**): Fluorescence images. (**D2**): Data analysis. (N = 6, ** *p* = 0.005; scale bar = 50 μm and 30 μm).

The cell membrane is an important barrier for biological cells to maintain integrity and homeostasis [23]. Studies have shown that the THz electric field mainly acts on the cell membrane [11]. Cell membrane topography analysis revealed significant alterations in the topography of SCC-7 cells after 2 h of 0.1 THz terahertz irradiation, exhibiting localized membrane protrusions (Figure 3A). Cellular ultrastructure analysis showed that SCC-7 cells’ volume decreased, the cells’ nucleus shrank, the cells’ bodies became irregular, the intercellular junctions decreased, and the chromatin condensed (Figure 3B). Cell membrane potential measurements revealed a significant decrease in SCC-7 cells following irradiation compared to the control group (Figure 3C, *p* < 0.01). Concurrently, calcium concentration analysis showed that the SCC-7 cells’ calcium concentration increased (Figure 3D, *p* < 0.01). Notably, L-929 cells showed no significant changes in membrane morphology or functional parameters under identical conditions. These findings collectively suggested that THz irradiation induced relatively specific bioeffects on SCC-7 cells through membrane-targeted mechanisms.

### 3.3. Terahertz Radiation Affected the Mitochondria of SCC-7 Cells

**Figure 4:**
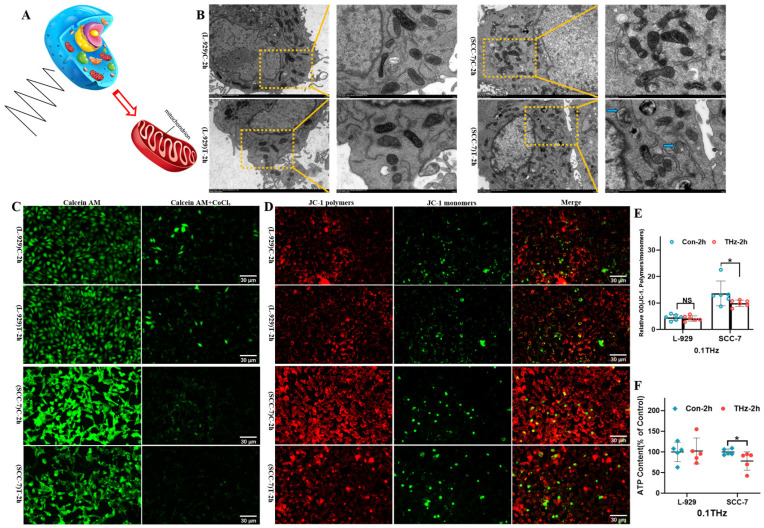
THz radiation affected the mitochondria of SCC-7 cells. (**A**): Schematic diagram illustrating THz irradiation of L-929 and SCC-7 cells (by Freepik, https://www.magnific.com/search?format=search&last_filter=query&last_value). (**B**): TEM images of mitochondria in L-929 and SCC-7 cells (blue arrow indicates mitochondria; scale bar = 2 μm and 1 μm). (**C**): Fluorescence images of mPTP in L-929 and SCC-7 cells (scale bar = 30 μm). (**D**,**E**): Fluorescence images and data analysis of mitochondrial membrane potential in L-929 and SCC-7 cells (N = 6, * *p* = 0.037; scale bar = 30 μm). (**F**): ATP content analysis of L-929 and SCC-7 cells (N = 5. * *p* = 0.016).

Mitochondria are not only the central site of cellular energy metabolism but also play an important role in the regulation of key physiological processes such as apoptosis [24]. To further evaluate the effect of THz irradiation on mitochondrial structure and function (Figure 4A), TEM was employed to observe the morphological changes in mitochondria. The results showed that some mitochondrial cristae in SCC-7 cells were disorganized and slightly swollen (Figure 4B). In terms of functional detection, mPTP analysis demonstrated that mitochondrial membrane permeability increased and fluorescence intensity decreased in SCC-7 cells after THz irradiation (Figure 4C). Mitochondrial membrane potential detection showed that the red-to-green fluorescence ratio was significantly decreased in SCC-7 cells (Figure 4D,E, *p* < 0.05), suggesting that THz irradiation might induce mitochondrial membrane depolarization. ATP quantification revealed a reduction in ATP content of SCC-7 cells following THz irradiation (Figure 4F, *p* < 0.05), indicating impaired cellular energy metabolism. In contrast, there were no significant changes in mitochondrial membrane permeability, membrane potential, ATP content, and morphological structure in L-929 cells. These results suggested that THz radiation had little effect on the mitochondrial function of fibroblasts.

### 3.4. Terahertz Radiation Caused Proteomic Changes in SCC-7 Cells

**Figure 5:**
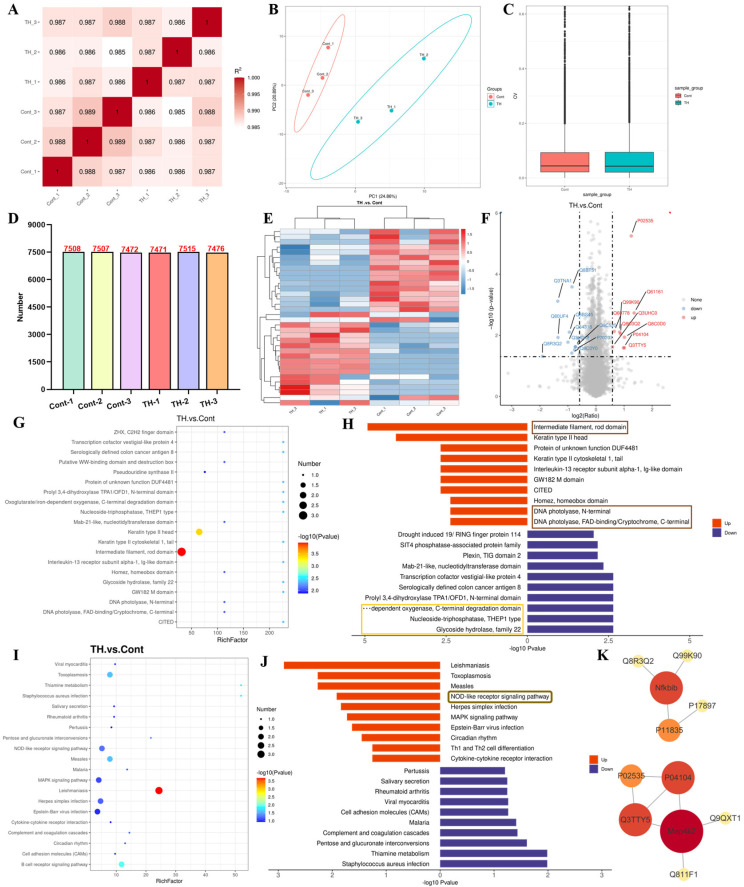
THz radiation caused proteomic changes in SCC-7 cells. (**A**): Correlation analysis. (**B**): PCA analysis. (**C**): CV analysis. (**D**): Protein histogram. (**E**): Heatmap clustering analysis. (**F**): Volcano map. (**G**,**H**): IPR analysis. (**I**,**J**): KEGG analysis. (**K**): PPI analysis.

To explore the underlying mechanism of the alterations in SCC-7 cells after 2 h of 0.1 THz radiation, proteomic sequencing was performed. A sample repeatability analysis was conducted as shown in Figure 5A–C. The results revealed that 34 differentially expressed proteins were identified among the 7403 quantified proteins, comprising 16 upregulated and 18 downregulated proteins (Figure 5D–F). IPR analysis indicated that the upregulated proteins were significantly enriched in “Intermediate filament, rod domain”, “DNA photolyase, N-terminal”, and “DNA photolyase, FAD-binding/Cryptochrome, C-terminal”. And the downregulated proteins were significantly enriched in “Glycoside hydrolase, family 22”, “Nucleoside-triphosphatase, THEP1 type”, and “Oxoglutarate/iron-dependent oxygenase, C-terminal degradation domain” (Figure 5G,H). KEGG pathway enrichment analysis further showed that the upregulated proteins were predominantly associated with the “NOD-like receptor signaling pathway” (Figure 5I,J). Additionally, PPI network analysis identified upregulated proteins Nfkbib and Mapk2 as key hub molecules (Figure 5K).

The above proteomic results revealed that THz radiation induced significant enrichment of mitochondrial-related signaling pathways and structural domains. Consistent with the experimentally observed declines in mitochondrial membrane potential and ATP levels, these findings suggested that THz radiation primarily targeted mitochondria, triggering functional impairments that subsequently led to energy metabolic disturbances and the activation of cellular stress responses.

### 3.5. Terahertz Radiation Induced the Release of Cyt c and AIF from SCC-7 Cells

Experimental and proteomic data indicated that THz radiation primarily acted on mitochondria. When the permeability of the mitochondrial outer membrane increased and the membrane potential decreased, Cyt c was released into the cytoplasm, initiating apoptosis. Meanwhile, AIF translocated from mitochondria to the nucleus, where it activated endonucleases and induced nuclear DNA fragmentation. Furthermore, the release of Cyt C might interfere with the electron transport process, leading to the accumulation of ROS and triggering oxidative stress [25,26,27,28]. Therefore, the levels of ROS as well as the expression of Cyt C and AIF were measured.

**Figure 6:**
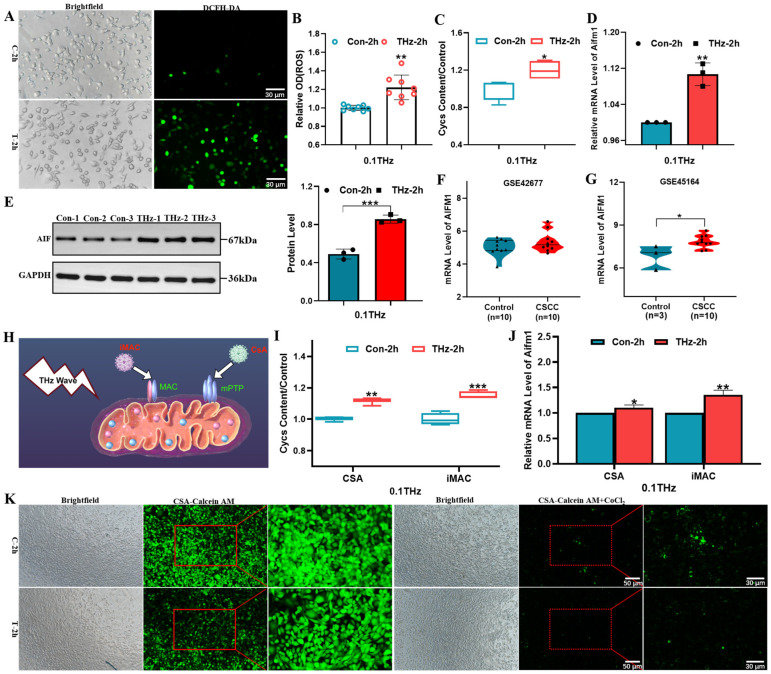
THz radiation induced the release of Cyt c and AIF. (**A**,**B**): Fluorescence images and data analysis of ROS levels in SCC-7 cells (N = 8, ** *p* = 0.002; scale bar = 30 μm). (**C**): Analysis of Cyt c levels in SCC-7 cells (N = 4, * *p* = 0.043). (**D**): QPCR analysis of Aifm1 gene (N = 3, ** *p* = 0.002). (**E**): Western blot analysis of AIF protein (N = 3, *** *p* = 0.001). (**F**,**G**): AIFM1 expression in the GEO database (* *p* = 0.015). (**H**): Schematic diagram of SCC-7 cells treated with CSA and iMAC by Figdraw (version 2.0). (**I**): Analysis of Cyt c levels in SCC-7 cells under inhibitor treatment (N = 4. ** *p* = 0.003 for CSA, *** *p* = 0.001 for iMAC). (**J**): QPCR analysis of Aifm1 gene in SCC-7 cells under inhibitor treatment (N = 3. * *p* = 0.036 for CSA, ** *p* = 0.002 for iMAC). (**K**): Fluorescence images of mPTP in SCC-7 cells under inhibitor treatment (treated cells with 5μM CSA and 5μM iMAC before irradiation, scale bar = 50 μm and 30 μm).

ROS content analysis demonstrated a significant elevation of intracellular ROS levels (Figure 6A,B, *p* < 0.01), and Cyt c content analysis showed increased release of Cyt c (Figure 6C, *p* < 0.05). QPCR and Western blot experiments confirmed that THz radiation could significantly up-regulate the expression of Aifm1/AIF (Figure 6D,E, *p* < 0.01 or *p* = 0.001). Meanwhile, analysis of two independent datasets from the GEO database indicated that in GSE45164, the expression level of AIFM1 in CSCC tissues was significantly higher than that in normal tissues (*p* < 0.05), while in the GSE42677 dataset, although the expression of AIFM1 also showed an upward trend, the change was relatively small (Figure 6F,G, *p* > 0.05). It is speculated that there may be some compensatory mechanism in CSCC patients.

To verify whether THz radiation facilitates the release of Cyt c and AIF by modulating mitochondrial channels (mPTP or MAC), CsA and iMAC were used to specifically inhibit their channels, respectively (Figure 6H). Under CsA-mediated mPTP inhibition, THz irradiation led to the opening of mPTP (Figure 6K) and induced the accumulation of Cyt c (*p* < 0.01, Figure 6I). At the same time, the expression of Aifm1 was significantly increased (*p* < 0.05, Figure 6J). Similarly, iMAC treatment significantly increased the levels of Cyt c and upregulated the expression of Aifm1 (*** *p* = 0.001 or *p* < 0.01, Figure 6I,J). These findings suggested THz irradiation activated compensatory mitochondrial apoptosis pathways through both mPTP-dependent and/or MAC-mediated mechanisms.

## 4. Discussion

Apoptosis is intricately regulated by various factors. Up to now, two distinct pathways for inducing apoptosis have been confirmed: the caspase-dependent and non-caspase-dependent apoptotic pathways. The former induces apoptosis primarily through caspase-3 cascade reactions, while the latter involves direct induction of apoptosis by the mitochondrial AIF [29,30,31,32,33].

This study confirmed that 0.1 THz radiation achieved inhibition of SCC-7 cells by targeting the cell membrane-mitochondria interaction network to trigger a non-canonical apoptotic pathway. The interaction between terahertz electromagnetic fields and cell membranes induced multi-level biological effects: at the physical level, THz electric fields altered membrane structures (e.g., localized membrane protrusions, cell shrinkage, intercellular gap widening, and reduced membrane potential), potentially causing membrane perforation effects [12,15] and Ca^2+^ influx, a process that closely matched the voltage-gated calcium channel (VGCC) activation threshold predicted by simulation models [13,14]. Studies have shown that 0.1 THz radiation can affect the dynamics of bound water molecules without inducing significant thermal effects, thereby triggering an order-disorder phase transition in membrane lipids and altering cell membrane fluidity [34]. Hu et al. [35] similarly reported that exposure to radiation at this frequency altered membrane permeability, providing additional evidence for its functional impact on the cell membrane. At the molecular level, calcium imbalance induced mitochondrial cascades, including disorganization of the cristae structure, mitochondrial membrane depolarization, and inhibition of ATP synthesis. Concurrently, the opening of the mPTP and MAC led to a burst of ROS, and enhanced the release of Cyt c and AIF, thereby establishing a caspase-3-independent apoptotic pathway. This phenomenon was aligned with previous studies, which have demonstrated that 0.1 THz radiation could impair mitochondrial structure and function, subsequently affecting the expression levels of related genes and proteins. These findings further support the critical role of THz radiation in modulating the mitochondria-mediated non-classical apoptotic pathway [36,37,38].

But this effect showed characteristics that varied significantly across cell types: under the same irradiation conditions, L-929 cells displayed no significant alterations in membrane or mitochondrial structure and function. This response may be attributed to differences in dielectric properties between cell types [39,40,41,42]. Studies have shown that, compared with fibroblasts, cancer cells exhibited higher dielectric constants [43,44,45]. Furthermore, cancer cells tend to carry greater negative electricity charges than normal ones [46,47]. In addition, cancer cells are typically irregular in size and shape, possess an irregular membrane morphology, have a relatively small cytoplasmic volume, and display irregular nuclear shapes [48,49]. These biophysical and morphological differences may render cancer cells more susceptible to THz radiation. Moreover, relevant studies have demonstrated that THz radiation did not significantly affect the DNA structure, morphology, proliferation, or migration ability of fibroblasts. Additionally, no significant changes were observed in intracellular reactive oxygen levels or the cytoskeletal structure [50,51,52]. These findings further supported the conclusion that THz waves may exert a selective effect on different cell types.

## 5. Conclusions

This study systematically evaluates the biological effects of 0.1 THz radiation on L-929 and SCC-7 cells. The results show that THz waves exerted significant cytotoxicity on SCC-7 cells, leading to abnormal cell morphology, membrane potential depolarization, and intracellular Ca^2+^ concentration imbalance. The structure and function of mitochondria were also markedly impaired. Omics analysis further confirmed that THz radiation primarily targeted mitochondria, causing metabolic dysfunction and activating cellular stress responses. Additionally, the ROS-mediated oxidative stress pathway was activated, promoting the release of AIF and inducing apoptosis in SCC-7 cells through a non-classical caspase-3 pathway. Notably, L-929 cells exhibited no significant changes under identical irradiation conditions, suggesting that THz radiation may be selective toward cancer cells. These findings revealed a potential mechanism by which THz waves induce apoptosis in cancer cells via the mitochondrial pathway, providing a theoretical basis for their future application in cancer therapy.

However, as a key regulatory factor in the mitochondrial stress pathway, the mechanism by which AIF contributes to THz wave-induced apoptosis in SCC-7 cells still requires further investigation. And future studies should also systematically examine the effects of a broader range of THz wave frequencies on cellular behavior, aiming to clarify the frequency-dependent variations in cellular responses. Moreover, future studies are recommended to use animal models of skin cancer to verify the biological relevance of in vitro findings and their potential translational value. To comprehensively assess the selectivity mechanisms and safety of THz wave exposure, it is also suggested that the experimental scope be expanded to include other types of skin cancer cell lines as well as normal skin cells. This will help systematically compare the differential responses of various cell types to THz wave exposure, thereby providing a more solid experimental basis for the application of THz waves in the biomedical field.

## Data Availability

The data used in this study are publicly available in the Gene Expression Omnibus (GEO) database: GSE42677: https://www.ncbi.nlm.nih.gov/geo/query/acc.cgi?acc=GSE42677 (accessed on 17 March 2026), GSE45164: https://www.ncbi.nlm.nih.gov/geo/query/acc.cgi?acc=GSE45164 (accessed on 17 March 2026).

## Supplementary Materials

The following supporting information can be downloaded at: https://www.mdpi.com/article/10.3390/cells15090810/s1. Processed gene expression tables (Table S1 and Table S2) derived from the public GEO datasets GSE42677 and GSE45164 are included in the Supplementary file accompanying this manuscript.

## Abbreviations

THz	terahertz
CSCC	cutaneous squamous cell carcinoma
AFM	Atomic Force Microscope
TEM	Transmission Electron Microscope
mPTP	mitochondrial permeability transition pore
ROS	reactive oxygen species
Cyt c	cytochrome c
AIF/Aifm1	apoptosis-inducing factor
qPCR	Quantitative real-time PCR
CSA	Cyclosporine A
MAC	mitochondrial apoptosis channel
GEO	Gene Expression Omnibus
PBS	phosphate-buffered saline
PI	propidium iodide
HBSS	Hanks’ Balanced Salt Solution
RLU	relative light units
IPR	InterPro
KEGG	Kyoto Encyclopedia of Genes and Genomes
HSP70	heat shock protein 70
HSP90	heat shock protein 90
IRB	institutional review board
OAP	off-axis parabolic
NS	not statistically significant
VGCC	voltage-gated calcium channel
